# What makes a Grand Slam champion? Early engagement, late specialization and timely transition from having fun to dedication

**DOI:** 10.3389/fspor.2023.1213317

**Published:** 2023-07-21

**Authors:** Mario Oršolić, Petar Barbaros, Dario Novak

**Affiliations:** ^1^ Faculty of Food Technology Osijek, Josip Juraj Strossmayer University of Osijek, Osijek, Croatia; ^2^ Faculty of Kinesiology, University of Zagreb, Zagreb, Croatia

**Keywords:** tennis, specialization, success, development path, interview, reflexive thematic analysis

## Abstract

In order to provide adequate support for young tennis players, it is important to understand the development path and factors which can have a positive or negative impact on the development of a player. The aim of this research was to determine the factors that affected sports specialization in tennis by means of qualitative analysis (early, optimal, or late). As well as to deepen the knowledge around the phenomenon of specialization in tennis among players of differing success levels through their personal experiences, in order to contribute to a better understanding of their specialization. The authors interviewed 30 tennis players of differing levels, including 10 participants from the most elite level (Grand Slam champions), exploring their contrasting experiences of development. The data set was analyzed using reflexive thematic analysis. Three topics were generated, which included the following: engaging in sport, decisions, and time of specialization. The obtained results offer a stimulus to reflect on numerous aspects of athlete development.

## 1. Introduction

There is an increasingly frequent discussion, both in the academic and sporting world, around the best pathway and age for children with a high potential for success to begin participating in sport (1). Uneven development paths experienced by athletes from sports in which top-level performance takes place before or after an athlete has fully matured, may imply various patterns of support by the parents, coaches, and peers during the development of an athlete. For example, in some sports, top-level performance is achieved at an adult age (2, 3). In order to provide support to young athletes, it is important to understand the development path and factors which can have a positive or negative affect on the development of their specialization, as well as to distinguish between athletes who achieve highly, as well as their less successful colleagues (4–7).

In scientific literature, many different viewpoints have been researched and presented. One point of view emphasizes the importance of a high level of specialization in young athletes (e.g., national team level and above) for the later achievement of high-level senior performance, whereas another point of view suggests the limitations of high-level specialization among young athletes for later senior high-level performance (6). For this reason, the question arises whether an early start in sports specialization is a prerequisite for later success in sport. A large number of previous studies indicate that early sports specialization is not a prerequisite for success, and that it can actually even hinder long-term achievement in certain sports (6, 8–18).

Despite the fact that early specialization in sports is increasing, recent studies found that late specialization (described as specialization after the age of 12) is more common among top-level athletes (19). Opposite to early specialization, elite athletes in multiple sports tend to start with more intensive training later on in their adolescence (20). In a study of elite and near-elite Danish athletes with the average age of 24,5 (athletics, weightlifting, cycling, rowing, swimming, skiing), the results showed that the elite group started with intensive training at a later age, as well as spending fewer hours practicing their main sport up to the age of 15 when compared to the near-elite group (21). For most sports, it is more probable that early diversity (diversification) shall lead to success (21–30). Current data on the sports specialization of elite, professional, and Olympic athletes is mostly retrospective and consists of survey-based evidence, according to which a majority of sports show better performance following the multi-sports engagement of young athletes. While additionally, sports specialization among young athletes is also associated to an increased risk of injuries among athletes at the highest of levels (31). Present guidelines for youth participation in sports greatly differs within and between sports, and there is lack of consensus on how young athletes should train. Other aspects of participation in sports also need to be regarded, as they can also contribute to the problem of sports specialization in young athletes (32). However, many parents and athletes believe that early specialization is the best method for becoming a top-level athlete (19, 33–35).

On the other hand, in a study overview by Mosher et al. (36) they found that there is explicit evidence that early specialization is harmful and should be avoided in any context. At the conclusion of the study, it was determined that without a consistent definition of early specialization, it is difficult to conclude on such a level of harmfulness of early specialization for young athletes as it is claimed by numerous organizations (e.g., American Orthopedic Society for Sports Medicine, American Academy of Pediatrics, International Society of Sport Psychology, National Association for Sports and Physical Education). Some defined early specialization as “year-long intensive training in one sport with the exclusion of other sports” (15), whereas others defined the beginning of early specialization as “the period when an athlete determines one sport as more important than another” (37). Hendry and Hodges (38) consider an increase in the number of training sessions per year as a key marker of early specialization, while Baker et al. (39) indicate early age and early inclusion into competitive sport as key parameters of early specialization.

There are presumptions among coaches, whereas the literature is not persuasive, as to the optimal starting age for beginning with tennis training that would allow for future success, given that tennis is a complex skill-based sport, usually characterized by long careers and with many top-level players actively playing even in their thirties (40–43).

Some coaches claim that many top-level professional tennis players started training as early as at the age of four, as well as that it is important to start early in order to ensure a satisfactory level of acquiring technical tennis elements (44). Further research by Li et al. (45) indicated that 75% of the top 300 players started playing tennis between the ages of 3 and 7, whereas 21% began playing between the ages of 7–10, and 4% started later, between 10 and 13. With regard to specialization, a study by the American Tennis Association showed that 70% of athletes specialized by the age of 10 years (46). However, a study by Carlson (24) concludes that elite tennis players specialized later and trained less than their near-elite peers between the ages of 13 and 15, but that they significantly increased their training after the age of 15. Result-based talent identification forces athletes to specialize in their sports at a younger age. Tennis experts suggest that on-court results should not be utilized as the only predictor of later success, particularly before puberty (47–49).

On the basis of all the above-mentioned, the conclusion can be made that it is important to focus research towards what athletes emphasize as relevant for their career to be successful, as such an approach has not been previously implemented in the creation of individual sporting careers, nor has the personal experience of one's own sports career been assessed in an appropriate manner. In view of the defined research problem, the following research question has been raised: which factors do tennis players of different levels of success perceive as key for optimal specialization in tennis? Therefore, the purpose of this study is to deepen the knowledge around the phenomenon of specialization in tennis among players of differing success levels by means of their personal experiences, in order to contribute to their better understanding, as well as to improve the process of creating individual sporting careers. In accordance with the mentioned purpose of this study, the aim is to provide an in-depth qualitative description of the development path of tennis players, as well as how different development paths affected specialization in tennis (early, optimal or late). The secondary purpose of the study is to collect enough data to share valuable information for parents, coaches, and junior tennis players. In layman's terms, which key decisions have former male Grand Slam singles players made during their junior careers that helped propel them to winning a Grand Slam or multiple Grand Slams?

## 2. Materials and methods

### 2.1. Philosophical perspectives and design

In this study, thematic analysis was used as a qualitative descriptive approach. Thematic analysis as an independent qualitative descriptive approach is mostly described as “a method for identifying, analyzing and reporting on patterns (themes) within data” (50, 51). Given that this study was focused on understanding the perspective of different development paths of tennis players during their adolescence, the qualitative descriptive approach was considered as appropriate. This study was positioned within the Interpretivist Paradigm as well as following Subjectivist Epistemology. The rationale for the approach was understanding the complexity of personal experiences of the tennis players, on the basis of which data shall be produced regarding differing levels of success among tennis players. On the level of content, this implies focusing on understanding what a certain life situation or problem meant for an individual player, as well as what can be used to enhance individuals' development paths of future players who are hoping to achieve success in tennis. By studying the beginning of sports specialization in tennis players, this study aims help identify tennis players who started with sports specialization either early or late on in their life. In this way, specific strategies and interventions within tennis practice can be created and implemented which could facilitate achieving a more adequate method of specialization in tennis. By determining the beginning of sports specialization, this study shall assist in producing an optimal approach for including children into the process of sports specialization.

Upon taking into consideration the aim of the study, male tennis players of differing levels of sporting success participated in this research. The selection method adheres to a specific approach for biographical studies which includes all participants who best correspond to the research questions and from whom the most valuable information shall be obtained in response to these questions (52).

Consequently, 30 players (aged between 25 and 50 years) selected by deliberate sample were interviewed. Eight of the participants are still active players, whereas 22 of them had finished their competitive careers. The research participants were divided into three categories: (1) Grand Slam winners (*N* = 10), i.e., tennis players who won at least one of the four Grand Slam tennis tournaments (Australian Open, US Open, Roland Garros or Wimbledon), (2) players ranked between 150 and 300 on the professional men's tennis rankings (*N* = 10) [according to the official website of the Association of Tennis Professionals (ATP) or who were at one point in their career ranked in that position], and (3) senior male tennis players of national rank (*N* = 10) (ranked between 1st and 10th place on the national ranking list or between 500 and 1,500 on the ATP ranking, i.e., who were at one point in their career ranked in one of these positions). All of our participants tennis careers took part between the time period 1990 and 2022.

### 2.2. Methods and data collection

In this research, a qualitative methodological approach was used by implementing biographical studies by means of collecting life stories from tennis players of differing levels of sporting success.

The participants, who meet the above-mentioned criteria, were invited to take part through direct personal contact. In order to ensure optimal personal context of the data, it was extremely important for each participant to distinctly relay personal experience to key phases which relate to their own careers (4, 53). It had previously been shown that such an approach increases the accuracy and authenticity of memories (4, 54) and had been an important step in overcoming certain limitations of retrospective remembering. This is a specific and key aspect for this type of data collection by ensuring the remembrance of each participant's personal experience related to a specific life period. Each question in the interview guide was open-ended, which resulted with a range of responses that were meaningful for each single participant.

All interviews were conducted by an individual educated in implementing such procedures, who is also a coach with extensive experience on the ATP tour. The above-mentioned circumstances allowed for a more comfortable atmosphere during the interview and sincere replies in communication with the players. Prior to conducting the interviews, a selection of the participants took part in pilot interviews. The pilot interviews greatly contributed to the increase of the research quality-level of this study, as they resulted in recognizing the need adapt certain questions or other methods which did not elicit proper responses or enable the researcher to receive a rich set of data (55). All interviews were transcribed verbatim, i.e., recorded according to what was said. Each interview lasted between 50 and 70 min, with an average duration of 60 min. The interviews where then checked with the co-authors, which enabled the head interviewer to rehearse and improve the interview process, including the comprehensibility and clarity of the questions, use of more accessible language for the participants, as well as the effective use of probes for elaboration and clarification (56–59).

Data collection was organized in three phases by applying the semi-structural interview method. The first phase included establishing contact with the potential participant via telephone call and email. The second phase referred to explaining the procedure, purpose, and topic of the research to the participants. The third phase included the implementation of the interview with the participant. The interviews were conducted in quiet and isolated places in order to prevent environmental factors (noise, light, bad weather) from affecting the participant's responses. A default interview structure was prepared in advance, however questions were open ended in order to gain individual insight into each participants different experiences. The interviews were conducted up until the point when the research topic was exhausted, i.e., as long as conversation reflections did not start repeating.

The interviews began with a detailed explanation of the aim and purpose of the research, which was then followed by the participants' providing their names and age, as well as a short introduction of their development path in tennis. The mentioned process facilitated the follow-up questions and transcriptions, as well as served as a type of icebreaker. After this initial phase, the interview was continued with questions regarding the specialization process of the tennis player. The problem questions within this study included: at what age did the player start engaging in sports?, at what age did the player begin training?, was tennis the only sport the player trained in? (if not, which other sports), did the tennis player train in a small or big club?, does the tennis player think that young players should immediately start training at top-level camps (clubs) in order to achieve elite-level results?, at what age did the tennis player start with more intensive tennis training?, does the tennis player consider that children should start with specialization in tennis as soon as possible?, if the tennis player could go back to the beginning of his career would he change something about the specialization process?, would the tennis player start earlier or later with more intensive tennis training and why would the tennis player start earlier/later with more intensive training?. The participants were asked to elaborate on their response to each of the questions.

Considering the subjective and individual nature of the research topic, each participant was asked an additional set of questions at the end of the interview in order to gain a deeper understanding of the sports path that tennis players go through. The questions at the end of the interview were asked with the aim to extract additional information, such as: what does success in tennis represent for each player?, which factors determine success in tennis?, is genetic heritage important for success in tennis, or are external conditions more important?, what differentiates successful from less successful tennis players?, and if there is something the player wanted to add or further explain regarding a previously discussed topic if a certain question was not asked or in case the tennis player was not able to fully respond in that instance.

### 2.3. Ethics

All participants agreed to take part in the study and gave prior informed consent, while the research was approved by the home institution of the second and third researchers and co-authors.

### 2.4. Data analysis

For the possibility of later transcription and interview analysis, all interviews were recorded with a tape recorder and video camera, which was communicated to the participants prior to the start of any said recording, and they were required to give their consent. Anonymity was guaranteed to each participant, as well as confidentiality of any personal information. Participants were allowed to remove themselves from the study at any given moment. All interviews were transcribed verbatim, i.e., recorded according to what was stated. The reflexive thematic analysis approach was used, which was developed by Braun and Clarke (60), for interview analysis, which comprises of six separate stages and is implemented inductively, so that the statements of the participants are coded with the aim to summarize them at the level of explicit meaning. The first phase comprised of interview transcription, which is deemed a good method for familiarizing oneself with the obtained data. The mentioned phase also included several readings of interview transcripts and the identification of data segments which contained significant information. The second phase consisted of producing initial codes from the data. Several potential topics were coded that were regarded as interesting and relevant for further research. The third phase involved sorting different codes into possible topics and comparing all relevant coded data extracts within the identified topics. The fourth phase consisted of a two-level review and refinement of topics, as follows: review at the level of coded data, and coding of additional data within topics which was omitted during the previous coding phases. The fifth phase was the definition and naming of the topics, during which the chosen topics were further refined. The final sixth phase consists of producing a report having finished with fully developing the topics.

## 3. Methodological rigor

Thematic analysis is a qualitative research method for identifying, analyzing, organizing, describing, and reporting on topics contained within a data set (50). Braun and Clarke (50) state that thematic analysis is a useful method for examining the perspectives of different research participants, pointing out similarities and differences, as well as generating unexpected insights. A series of steps was introduced in order to increase data credibility (57, 61). Conducting pilot interviews (and using the same interviewer the entire time) assisted in increasing the consistency of the interviews. The interviewer's experience (identical as in the pilot interview) as a coach with a rich background on the ATP tour procured trust and a comfortable atmosphere during the interview. All interview transcripts were delivered to the participants for their verification, allowing them to add, delete, or revise any data they considered as wrong in relation to their communication during the interview (56). Furthermore, the third author subsequently contacted the participants by telephone in order for them to confirm their statements and discuss the study findings. All of these activities confirmed to the authors that the participants were properly instructed for the research involvement. All of the participants verified their transcripts. All three members of the research team participated in joint meetings and collectively took part in the analysis of the obtained data. The mentioned meetings included detailed discussions on the standout topics. There were some uncertainties in relation to some of the topics, however, following repeated meetings and detailed analysis, an agreement was reached in order to present the obtained data as accurately as possible. The final coding scheme was agreed in the course of the above-mentioned discussion and analysis. This important process contributed to data credibility, ensuring the interpretative validity, and minimizing the risk of individual research bias (62).

## 4. Researchers' reflexivity

I have personally (third author) experienced the misunderstandings of my coaches and my surroundings in tennis, as a result of which my wrong decisions arose from not understanding the path experienced by top-level athletes with the aim to achieve the best possible results. For this reason, I decided to terminate my sports career and enroll into college. At college I specialized in tennis and started working both as a tennis and a physical conditioning coach. Athletes' limitations on the path towards achieving top-level results became the subject of my research interest. It was my own personal sports experience that created this desire to study the mentioned area. Both the first and second authors (also former tennis players and coaches) took part in the data collection process, as they also ended their sports careers and engaged in researching athletes' success. Conducting reflexive discussions with both mentioned co-authors assisted me in elaborating on my presumptions, as well as in remaining focused on the experiences of the participants, instead of my personal situations. All of the above-mentioned is reflected in the subjectivist epistemological position of this paper.

## 5. Results

In keeping with the aim of this study, data analysis resulted in a large amount of relevant information which had an impact on the development of the careers of the athletes involved. The data analysis aimed to include quotes by the participants within each question or sub-question. Stories of the participants from their childhood onwards were presented with the aim of demonstrating how different events and experiences in key developmental moments formed their sporting careers. The analysis resulted in seven sub-topics that were presented as part of three main topics: (I) engaging in tennis, (II) decisions, and (III) time of specialization.

With the aim to facilitate the tracking of quotes, the participants were marked according to their category of success. Grand Slam winners were tagged as Level 1, participants ranked between position 150 and 300 were Level 2, while participants ranked between position 500 and 1,500 were Level 3. The second number of each mark represents the sequential order when the participant was interviewed within his category.

### TOPIC 1. Engaging in tennis

This topic represented the different ways in which the participants described their beginnings in tennis. Within this topic, there were three sub-topics: important people relating to an athlete choosing tennis, impact of additional activities, and growing up in different environments.

#### Important people relating to an athlete choosing tennis

An early beginning in playing tennis was found in almost all participants at the third level of sporting success. This mostly included tennis lessons, mini tennis, and playing in groups, with training sessions being once or twice per week. However, two participants from the third level of success stated that their first contact with tennis took place when they were three and four years old:

“I think I was four years old, and somewhat more seriously probably at the age of six. I don’t remember that, but my parents told me so.” *L3/9*

Participants at the second level of sporting success, in comparison with their less successful colleagues, had a similar early beginning (at the age of six or seven):

“I started when I was six and a half years old, before I started first grade at school. It was tennis school, mini tennis.” *L2/7*

While a similar early start characterized participants at the second and third level of sporting success (at the age of 6 or 7 years old), most Grand Slam winners had a somewhat earlier start (aged 4 or 5). In the beginning, these were training sessions once per week, and when the coaches noticed their talent, they started training twice per week and were included into team training with older players. They described their early beginning in tennis as follows:

“I started when I was five years old and I played only one day per week, on Saturdays.” *L1/1*

“So I started playing tennis when I was really, really young. I was very small, almost still in diapers. When I was four years old, at home I was always hitting a ball against the door of our apartment.” *L1/2*

“I began playing tennis when I was four, three or four, early. When I was really young, maybe one and a half years old, or one, as soon as I could crawl, I had a wooden tablespoon for soup and a small ball, and I would hit the ball and then crawl to catch it, that is how I started.” *L1/3*

The participants hereafter described the process of choosing tennis, i.e., who initiated the choice to be precisely tennis. With regard to them starting to play tennis, the participants recounted their experiences in detail while reminiscing and illustrating various individual events, whereas their beginnings in the sport and how they ended up specifically in tennis, they consider a be a combination of circumstances.

Participants at the third level of sporting success chose tennis as a result of multiple reasons, and some of them stated that their brother or sister already played tennis:

“My sister started tennis before me, and then, along with her, I also tried it. I went to her training without any obligations and then, in the end, I liked it.” *L3/2*

One of the participants reported that he was the initiator in deciding upon tennis:

“I saw tennis on the TV and then I told my father that I wanted to play.” *L3/4*

Another participant at the third level of success stated that his PE teacher played a considerable role in his choosing when he contacted his parents:

“The PE teacher was a private coach, and he asked my parents if they wanted their child to try playing tennis. And that was how it started, and then I played my first tournaments.” *L3/5*

Some of the participants from the third level of success said that they chose tennis mostly because the tennis courts were nearby their house or because they saw their father playing tennis:

“I lived 150 meters away from the tennis courts and that's how I ended up in tennis, and not, for example, at a football stadium which was farther away.” *L3/3*

“My father was banging the tennis ball against the wall, and my sister and I were running around him and shouting: ‘Let me try it, let me try it!’. And then we took that racquet in our hands and banged the tennis ball against the wall. Six months later, we enrolled in a tennis school.” *L3/6*

It is interesting that among participants at the second level of success, fathers played an important part in their child's enrollment:

“My father played tennis recreationally and applied for a coaches’ license. And so I played a little with him. I liked it and after that the racquet never left my hands.” *L2/9*

One of the participants at the second level of success described choosing tennis by emphasizing that it was exclusively his initiative:

“It was my initiative to play tennis. I also played football, and I was good at it, but I left it and went to play tennis. There was plenty of my initiative, and it is not often seen nowadays that someone would simply take a racquet and tennis ball and go and hit it against a wall.” *L2/4*

Some of the Grand Slam winners pointed towards fathers as key individuals:

“My father started playing at a club. He joined in and started playing tennis. He then brought me and my brother, who was five years older than me, with him to play at this club. So yes, I followed my father, joined the club, and enrolled in a tennis school.” *L1/1*

“Tennis was a new sport here, and my uncle was the head of some tennis club in Germany. That is how my father took me and my older brother to training, and so I started training tennis.” *L1/7*

#### Impact of additional activities

Additional activities turned out to be an interesting topic in which a significant difference can be observed among the players of the three different levels of success. For example, for participants at the third level of success, tennis was the only sport they engaged in, while participants at the second level of success also showed interest in other sports. Despite being interested in other sports, they were nonetheless not included into training at organized sports clubs for these other sports.

When they started playing tennis, participants at the third level of success did not demonstrate interest in playing other sports at organized clubs.

“I was at tennis the entire day, at that point I trained tennis for six, seven hours.” *L3/10*

However, during their developmental years, participants at the second level of success also showed interest in other sports, but they pointed out that their attention was still directed towards tennis:

“Yes, there was also football and handball, but that was all on an amateur level. I mean, there was no thinking about any other sports for me as soon as I felt that this was it.” *L2/1*

During their developmental years, Grand Slam winners also showed a liking for other sports. In addition to tennis, they also actively trained in other sports, whilst their attention was equally directed both towards tennis and the second sport that they were involved in. It was not until their later years that they focused solely on tennis:

“At first my father wanted me to play two sports, tennis, and ice hockey. And so I did that for one or two years, but then I concentrated only on tennis.” *L1/3*

“At sports school I learned athletics, cricket, field hockey, basketball, and different kinds of sport, so that I developed as an athlete practically before I developed as a tennis player.” *L1/5*

#### Growing up in different environments

Participants feelings towards the challenges of growing up in a smaller towns or larger cities when related to sport showed a high level of awareness on the certain advantages and disadvantages that could be present. The participants believed that the size of the city definitely affected the beginning of sports specialization.

However, growing up in a smaller town meant athletes did not have too much of a choice, i.e., the option of training in other sports, directed them towards early specialization. A bigger city offers more possibilities in terms of a more versatile development, but also a wider selection in choosing more expert training teams. One of the participants at the third level of success who grew up in a smaller town stated the following:

“In a big city there are certainly more quality-level coaches, and there are certainly more options if you are looking for a private coach. It is a fact that you have a wider choice for playing other sports, and at a certain point you can better decide on what better suits you.” *L3/4*

Several participants at the third level of success pointed out that leaving the smaller town for a bigger city made them feel uneasy and less safe:

“What was a huge disadvantage for me when I was leaving for bigger cities was that I felt uncomfortable or perhaps less safe because in my smaller environment I felt comfortable.” *L3/3*

Participants at the second level of sporting success described how growing up in a smaller or bigger town affected the beginning of sports specialization. Likewise, they stated that with consideration to the environment they grew up in, they did everything they could to benefit from it, as well as that some tennis players, regardless of the limitations of growing up in a small town, managed to get to the international level:

“Yes, I do think that the size of the town does affect specialization. In environments where there is only one sport, then everyone plays that one sport.” *L2/3*

“Some of my colleagues at a certain point in their growing up, just like me, when they turned 14, 15, 16 years old, started training somewhere else because this was a too small environment for them.” *L2/5*

In addition to this, they also stated that growing up in a big city offers a wider set of possibilities, for example, tennis clubs, the option of sparing with better players, and competitions, etc.:

“I think that bigger towns offer more when it comes to the conditions. But I think that in the end all that is not as crucial as, in my opinion, that there is not really any education for those children, coaches and parents who work with those athletes during those phases.” *L2/9*

Grand Slam winners described growing up in smaller or bigger towns to be less relevant for their advancement, however, that this aspect also depends a lot on one's character. They emphasized that between the ages of 8 and 14, the most important aspect is to train and have fun:

“I think that it depends on someone's character, as well as on their situation at home. So, I am not an overly social person, and that's why I think that it wouldn’t be good for me if I had been growing up in the city. But I do know some players from my youth, who grew up in the city and they were good players when they were younger, but then, when they were 13 or 14 years old, they started going out a little, and having other interests, and in cities there is a lot going on. There is nothing interesting happening in the village.” *L1/3*

One Grand Slam winner believed that growing up and training in a smaller town was an advantage for him and that because of this, his attention was directed solely towards tennis:

“So, the attention, perhaps, that you receive in a smaller town is greater, and you can train much more. However, at the same time, I was really lucky because in my club there were players who were in the top 7 in the world, and there were also other players, I would say between positions 120 and 400. And that is why I always had excellent sparring partners, you know, partners with whom I hit tennis balls with. And players who could teach me how to improve my game. That's why I couldn’t have been in a better place, and have a better environment for growing up as a tennis player.” *L1/2*

### TOPIC 2. Decisions

The second topic of this research piece concentrated on the decisions that guided the subsequent tennis career of each participant. This topic contained two sub-topics: the development path, and from having fun to transitioning towards a more dedicated training process.

#### Development path

All three categories of participants elaborated on both the positive and negative sides of their experiences within their own tennis development. What significantly characterized participants at the second and third level of sporting success was a premature narrow specialization, as well as the fact that at some point, they lacked competition. The majority of the participants from the second and third level of success pointed out that if they could go back in time, they would build their tennis story on the principle of broadness, where they would partake in more international events to gain more experience as opposed to staying in their home country and competing in local tournaments.

“I lacked, however, some of that competitive experience. I would add that perhaps I should have competed more in some international tournaments to get a broader perspective.” *L3/2*

It is interesting that going to world renowned tennis academies was marked as one of the key decisions in their sports careers that they perhaps should not have made. Participants’ perceptions were that staying at the best tennis academies was not the right path towards development for these players, and that they believe they would have achieved much more progress if they had stayed at home with a good coach.

“I was at one of the best tennis academies and I think that wasn’t the right path. I lost several years at the academy, and learned probably 10 to 15% of what I could have learned with a good coach, and a quality programme. I would’ve learned more in three weeks during the summer when I was at home, then I would’ve learned in six months at the tennis academy.” *L3/8*

As a reason for the inability to reach their full tennis potential, participants at the second level of sports success also mentioned their experience in going to world-renowned tennis academies:

“I consider that to be a mistake as I think that I had been more focused on some other things, instead of tennis. At home I would’ve surely been more focused on tennis than I had been at the tennis academies.” *L2/1*

They would postpone going away to tennis academies for a time when they would be ready to focus on nothing other tennis:

“I would change only that I would prolong another few years of being at home during that period between 13, 14 and 15 years old.” *L2/1*

Some of the participants stated that they did not have sufficient tennis skills and that they learned certain skills as late as at the age of 20, 21 and 22. The participants felt that by that point, they should have already known these aspects if they were to progress to a better level. They believe that their path towards success could not have been kept up by insufficiently competent coaches and the work that had been carried out in younger age categories:

“I think that we had coaches who only started and that it was them who learned from us. This is perhaps one of the reasons why among many players, and also in my case and many of my colleagues, a pronounced weariness takes place after a certain number of years if specialization is started too early.” *L2/5*

“In my case, I lacked this through the childhood years, a certain type of education, and here I mean things around tennis, such as some kind of physical work, i.e. physical conditioning training. What I would’ve liked was that I had the opportunity of someone educating me a little better and teaching me why you must stretch out, why you must run for condition, what that adds up to in the end.” *L2/9*

As positive example one of the Grand Slam winners pointed out was the moment they started to train with an elite group of players:

“The key moment in my career was when the federation put us in the group with older players. Three other top players in my age group were also there. We always trained together.” *L1/1*

*“*I was in luck, I ended up with a coach who also trained four national champions.” *L1/2*

Nevertheless, the most significant was the reaction of the Grand Slam winners to the support they received from their teammates. They considered the support to be the accelerant for their future success:

“We had much success with assembling teams as, you know, if he can win the championship, then I can also win the title because I won against him at last week's practice. You support one another and that's something that I think is extremely efficient. I this that's very successful all around the world.” *L1/2*

Settling down in a new environment and a new club was a challenge which was difficult to deal with even for Grand Slam winners:

“Allow me to explain one key moment in my career, and that crucial point was when I moved. Yes, that transition was pivotal for me. I wasn’t feeling good in the beginning. I didn’t like it, and I went home for the weekend every Friday afternoon for almost two years. That wasn’t easy for me.” *L1/1*

#### From having fun to dedication in the training process

In some parts of the interview, the participant also often mentioned dedication to the training process, thus the mentioned sub-topic proved to be extremely important in the segment of reaching their tennis potential demonstrated at an early age. The participants at the third level of sporting success demonstrated the opinion that they were not actually fully committed and enthusiastic in their training process:

“Perhaps the difference between me and the players who reached the top level is that they enjoyed it, while us who didn’t reach that level, we weren’t serious, we fooled around, etc. I wasn’t responsible enough. I would say that my training was excellent, however, it wasn’t always 100% as when there was someone with me. If it was a great day, then I would have a great practice, but if it wasn’t, then my performance would be at 60 or 70%.” *L3/1*

On the other hand, there were once again explicit qualitative differences between participants at the second and third level of success. The participants at the second level of sporting success were mostly characterized by a positive dedication, love for tennis and focus:

“Well I think that 100% dedication is important for success in tennis, and I mean really 100%. So, if you are willing to work, then it must be at 100%. There is no other option, only that. Dedication and arrogance. Arrogance, you must be really audacious to show your teeth and to realize that nobody can break you.” *L2/1*

“First you want to achieve certain goals, and that kept me going between the ages of 21 and 30. Although there were rough times, I had been injured, I wasn’t earning money, I had accomplished enough, but not too much. And then I made a career from the age of 30 until I was 40 years old.” *L2/2*

Despite the fact that they trained really hard and were focused on tennis, they felt that they were missing a certain detail to become even more successful. They emphasized that tennis is a specific sport and that for success, it was important to integrate a number of factors:

“I was really concentrating on my tennis, how to improve my strokes, but on the other hand, I wasn’t doing enough work on speed for my body. Therefore, that was one of the mistakes in the end. I had to be very careful, but earlier, between the age of 20 and 30, I surely wasn’t doing enough work.” *L2/2*

“Modesty is certainly my virtue, however this package, which is necessary for a professional player to get into the top 100, I didn’t have. I had 9 out of the 10 required elements (categories), but I didn’t have this one I was missing—perseverance.” *L2/4*

Grand Slam tennis players demonstrated exceptional dedication and love for tennis even in their earlier years:

“I had found my love for sport at a very early age. I was a fanatic when it came to tennis. I only wanted to play, and play, and train, I never had enough. Sometimes my mother and father had to drag me from the tennis court as it was enough, they said, and I had to go get some sleep and eat, get myself ready.” *L1/2*

Success and winning Grand Slam tournaments can be contributed to dedication, focus, and love for the sport:

“But I believe that the basic thing lies in what you really want. The basic thing is your commitment and your love for the sport you’re playing.” *L1/10*

### TOPIC 3. Time of specialization

The third research topic was oriented towards the time of player specialization. Within this topic, there were two sub-topics: targeted and more intensive training, and most optimal time for targeted and more intensive training.

#### Targeted and more intensive training

A large number of participants at the third level of sporting success pointed out that they started with targeted and more intensive tennis training very early on in their tennis career. They attribute their early beginning with more targeted and intensive training to talent, due to which they acquired faster initial tennis skills and for this reason, with their coach's initiative, they transferred from group to individual training:

“I didn’t stay for long in club training as it wasn’t working for me, so I, starting since I was seven or eight years old, trained everything individually.” *L3/1*

“I transferred from group to individual training around the age of eight, and that was early. I remember being better than the others in the group, so the coach took me to tournaments.” *L3/5*

However, some of the participants at the third level of success trained in groups the entire time, and they did not perceive the precise transition to more intensive trainings from group to individual sessions:

“Everything was mostly organized at the club-base level, club trainings, sparing partners, matches, etc. So I don’t really know about private classes (laughter).” *L3/4*

“To be completely honest with you, it was all a game to me. When did the exact transition take place, I don’t know, I can only say that I did not notice that there was any transition.” *L3/10*

Among participants at the second level of success, a later start of targeted and more intensive training is noticeable. Data analysis determined that the reason for this was playing another sport at a recreational level, which the participants at the second level of success equally loved. Below are their statements regarding the age at which they focused and trained more solely in tennis:

“Well, I started training more seriously when I was 14 years old, that is when I began playing tennis every day.” *L2/3*

“I started training more seriously at the age of 14, 15, and then at the age of 16, I went to Germany. I totally went into professionalism.” *L2/4*

The statements of Grand Slam winners are not in favor of early specialization, i.e., of prematurely focusing and training intensely in tennis, which is confirmed by the data analysis—as they themselves did not start too early with specialization:

“I started training more individually when I was perhaps 12, 13 years old, when I played at the national championship. At that point I trained almost every day. And it was not until later on that I started competing on weekends, both individually and in team competitions.” *L1/1*

“I played and trained in other sports as well, and then, when I was 15 years old, there was only tennis.” *L1/5*

“I began training more seriously and more intensively only after finishing elementary school, for sure not too early.” *L1/7*

#### The most optimal time for targeted and more intensive training

Below are some considerations of the participants regarding the most optimal time for starting with targeted and more intensive training (narrow specialization) in tennis. The participants believe that in sport today, and thus also in tennis, intensive individual training starts sooner than it did previously, and that this is not beneficial. Likewise, they elaborated on the fact that focus should be directed more towards technique, as well as that players should be slowly introduced into competitions.

Participants at the third level of success also maintain that children should not focus exclusively on tennis too early on in their life, however, this surely varies from one player to the other. On the basis of their personal experiences, participants stated that they would recommend that children train at club level, and that only occasional individual training is included.

“Today I would advise children and their parents to keep this sort of club programme until the age of 12 to 14 years old, however, also to introduce individual training once or twice a week.” *L3/2*

“In my opinion, narrow specialization should be introduced at that moment when the player has sufficient tennis broadness and when he's physically mature enough for it. I would certainly build the story on broadness, and in no case on narrow specialization in the sense of having many tournaments.” *L3/3*

Some participants believe that parents pressure their children and start with premature targeted and more intensive training due to the fact that they did not succeed in their own accomplishments, i.e., did not have a successful sports career themselves.

“The problem is when parents pressure their children and then the child decides something on force and is not sure of its choice.” *L3/1*

“Sometimes people who didn’t accomplish something when they were young, they ask that of their children, whereas the children perhaps aren’t ready and don’t want that, and then problems occur.” *L3/6*

Participants at the second level of success also reported that it is not a good choice to start with premature specialization. Equally, they believe that children should be directed towards engaging in as many sports as possible and with the main goal being to have fun.

“When you’re 15, 16 years old, you can play tennis, but before that, it's too early in my opinion. It's dangerous to specialize too early.” *L2/2*

“I think it's important to also train in another sport, such as athletics, football or any other sport, as long as you’re training something else.” *L2/10*

This type of more versatile beginning and later specialization was additionally emphasized by one participant for whom narrow and early specialization did not prove to be the optimal solution. The below quote confirms the thesis on why his friends quit sport early on in their life:

“I know a lot of children, my friends, who were in sport from the age of 1 to 12, or to the age of 14, and then they gave up on sport. I think that it's very important to start slowly.” *L2/3*

Statements from Grand Slam winners suggest that it could be beneficial for future aspiring players to develop their motor skills through engaging in other sports at a young age, as well as not to start too early with targeted and intensive training (narrow specialization). It is interesting that Grand Slam winners pointed out the following—that for someone to be a better and more successful athlete, they feel that it would be highly beneficial for players to play as many sports as possible, and their most optimal suggested time for targeted and intensive training was described in the following quotes:

“At a certain phase, if you want to be successful, very successful in a certain sport or to be excellent in something else, you must focus on a specific topic or one sport, depending on what it is.” *L1/5*

“I think that if you strain your body too much before it has completely grown up, there is a great risk of many injuries, which will then cause problems.” *L1/7*

“I don’t know, but I certainly wouldn’t play for three of four hours each day of the week. And you need to rest also, you know.” *L1/10*

## 6. Discussion

This is the first study which presents qualitative research factors that affected specialization in tennis among players at three levels of sporting success: (1) Grand Slam winners, (2) players ranked between position 150 and 300, and (3) players ranked between position 500 and 1,500 on the professional men's tennis rankings. Through thematic analysis, early specialization was identified as an important barrier to a successful tennis career. The accounts of successful and less successful players provided valuable insight into how their tennis careers initially developed, players' perceptions of how specialization impacted upon success, and the consequences of early specialization experiences for overall success.

Grand Slam winners described that when they started playing tennis at a very early age, it was characterized by a small number of training sessions per week, they also emphasized that tennis was not the only sport that they engaged in. Balyi, Way and Higgs (63) claim that at the purpose of the childhood development phase should be to instill the love for sports and encourage children to get involved in sport. Introducing children to a racquet and ball appropriate for their age group, improving hand-eye coordination, gaining confidence, learning various basic tennis moves—are all some of the goals which should be achieved during this period according to Lloyd et al. (64). Starting early is quite often correlated with success, and thus Li et al. (45) state in their research that some coaches claim how many top-level professional tennis players started as early as at the age of four, as well as that it is important to start early in order to acquire technical tennis skills. However, studies have shown that an earlier beginning and a greater volume of specific sports training and competition during adolescence is not necessarily correlated with greater success (65, 66). According to Roetert et al. (67), coaches have a significant role in terms of introducing young children into tennis and teaching the skills required for enjoying the game for the duration of one's lifetime. Coaches can surely play an important role in achieving a healthy, positive and educational surrounding which is suitable for proper skill development (68). Nevertheless, the inclusion of young children in sports greatly depends on the parents, as parental influence presents 57% at that age, whereas later on this important role is taken over by sports clubs and coaches. Parents have the greatest influence on the decision of a young athlete to play tennis (69).

Studies have shown that young children who participate in a large number of sports at a younger age have higher results in tasks of gross motor coordination, as well as a reduced risk of injuries in comparison to children who specialized in only one sport at an early age (70, 71). Without the opportunity to try out different sports during their childhood, it is less likely that young athletes shall acquire fundamental physical, psychosocial, and cognitive abilities which are relevant for long-term success in sports (72). By specializing in only one sport, athletes can reduce the need to participate in versatile sports activities throughout the entire year, which can in turn result in a decline of sporting skill development during one's lifetime (73).

The size of the city where an athlete is born can be a significant prerequisite for future sporting achievements (74). Studies quite often point out arguments which explain why it is more advantageous to grow up in smaller to middle-sized cities, as opposed to bigger cities. Despite the fact that big cities can offer children and young athletes better conditions, such as well-designed and equipped sports facilities and better coaching leadership, it appears that sports programs implemented in big cities significantly lack time, more personal athlete-coach relationships and an insufficient focus on the individual (75, 76). Combined qualitative reports provided by coaches and quantitative data analysis from various research pieces further support the idea that smaller and middle-sized cities, as opposed to big cities, are considered as a more suitable sports environment for children and adolescents (aged between 14 and 18) (77). For the above-mentioned reasons, training in a smaller town has its advantages, however, in order to achieve better sports results, after the age of 14 it is necessary to switch sporting surroundings from smaller to bigger cities.

The private sector in tennis offers highly professionalized training centers and academies which provide all-inclusive development paths for players, from talent identification to elite-level tennis (78). Tennis players advance through a talent development process to tennis clubs and national training centers or private academies (79). Data analysis revealed that the participants were not satisfied with going to tennis academies, and therefore Crespo and Over (80) believe that the key role is that of local-level tennis clubs, both in providing the opportunity to participate in sports, and providing modified sports programs and competition formats. Consequently, De Bosscher et al. (81) consider that the development of elite athletes, including success at national and international competitions, inevitably requires a strategic approach to the development of top-level athletes.

Having fun leads to dedication in the training process, thus this seemingly insignificant factor during training holds the potential of having a significant role in terms of ultimate success. Weiss et al. (82) presented results which found that having fun was the primary indicator of dedication in sports among 198 USA junior tennis players. Another study by Casper et al. (83), including adult tennis players in the USA, revealed that having fun and personal investment represented the most significant preconditions for dedication in sports. In an interesting research piece by Weiss and Neibert (84), the authors reported that the level of dedication among athletes can alter in the process of aging. The results of this study likewise showed that Grand Slam winners were more enthusiastic and dedicated to training, and also considered enjoying the game as a main factor for success.

There is no evidence that intensive training and specialization before adolescence is required for reaching elite-level status in individual sports such as tennis. The risks of early sports specialization include higher injury rate, greater psychological stress, and ceasing to play sports in early youth (85, 86). In a paper by Li et al. (45), the results showed that the majority of players started tennis between the age of 3 and 7, which indicates that tennis is a sport that children start playing early, which seems to be an important if the aim is to achieve success on a professional level, while such results are also confirmed by research in football implemented by Hendry and Hodges (38). Furthermore, a common characteristic of those who started late with more targeted and intensive training was that they also engaged in other sports as well (e.g., football, gymnastics, etc.). The mentioned research points to the fact that an early start in tennis and late specialization can also result in elite-level success (87). However despite this,, young athletes are increasingly getting involved in early sports specialization (i.e., intensive engagement in only one sport starting at early childhood) in order to gain an early advantage in the sense of developing skills (88).

The age at which young athletes should specialize is one of the most relevant discussions in youth sports today, whereas researchers from around the world have been debating this issue for decades (89). Determining the timely beginning for sport specialization in young athletes, and its effect on the further career of athletes, present the key finding of this study, which will assist in defining the optimal time for introducing children to tennis. The debate about specialization is grounded on the question when an athlete should specialize in a particular sport. According to Jayanthi et al. (20), sports specialization in tennis should be constantly ongoing. In the study by Schneider and Jayanthi (87), it was concluded that an early introduction and late specialization in tennis can result in elite-level success. It is interesting to recognize that all Grand Slam winners developed their career precisely in that manner. Sports specialization is recommended in late adolescence, as early specialization results in great risk of injuries, psychological stress and reduced participation in sport, which is definitely identified in this study and further confirmed in various other research pieces (19, 20, 36, 90).

It is precisely the experiences of Grand Slam winners that shows it is possible to reach the top of the game without specializing early. It looks like pre-adolescent specialization is not always a requirement to become an elite athlete. Moreover, later specialization may mean there is more chance of children choosing the sport that is best for them—not simply one that suited their physical predispositions better at a particular time, or was their parents' favorite (91). Based on the findings of this qualitative research, we might conclude that a hybrid approach—early engagement and later specialization—offer a prudent alternative to early specialization (91, 92). In the early engagement model, players are actively encouraged to accumulate sufficient hours in their main sport, in this case tennis, so as to not accumulate a large practice deficit relative to early specializers. But early engagers are also encouraged to continue playing as many sports as they like to gain the necessary sport-specific skills, as well as a broad range of physical and mental skills (91). There are no empirical studies on early engagement in tennis, however, there are some studies supporting this model in other sports (38, 93, 94).

A key strength of this study was that a substantial proportion of the sample were Grand Slam champions, which is uncommon in qualitative and sport science research. Studies often research less successful tennis players because they are much more accessible. There is different criteria for determining the sample size within qualitative research (60). The sample size containing sufficient information strength depends on the following: (a) aim of the research, (b) specificity of the sample, (c) use of the established theory, (d) quality of dialogue, and (e) analysis strategy (95). With a total of ten Grand Slam champions recruited in this study, the sample size met the recommendation for a qualitative research study (60). However, it is important to acknowledge that the research findings may not be generalizable to the entire population of male tennis players. Furthermore, the content quality of this study is demonstrated by the possibility of generalization, reliability, and versatility of the obtained data. We believe that the presented results provide an adequate incentive for speculation among coaches, parents, and any other members of sports staff regarding the proper manner of supporting young athletes.

Focusing exclusively on male tennis players presents the main drawback of this study, as it does not provide a complete picture on sports specialization in tennis. Future studies should certainly be directed towards researching personal situations and experiences among female tennis players. Upon insight into literature, one can notice a very small number of qualitative research studies on the personal events and experiences of athletes/tennis players at differing levels of sporting success, as well as studies on sports specialization in tennis in general, and thus this study provides a basis for other researchers in their future research within this field. There is a need to determine how other barriers influence tennis careers and impact levels of success in tennis. The inter-relatedness of other barriers may be important in understanding a holistic approach to improving success levels of younger tennis players, and for the measurement of success-related barriers. However, more research is needed to explore these inter-relations.

Based on the presented study results, it can be recommended that first introduction to tennis should take place early on in an individuals life, however, it should be directed primarily towards evoking the feeling of having fun. Furthermore, we can say that an early engagement in tennis should be followed by later specialization.

## 7. Conclusion

This qualitative study examines the experiences of three differing categories of tennis players. The players reflect on their perspective of their own sporting careers, and on how they experienced their development path prior to reaching a certain level of success. The results of this study clarified the difficulties that tennis players are faced with on their path towards success. The results also enable planning for the optimal time to start with sports specialization in tennis for future tennis players, coaches, and other sports staff.

In the process of this review, topics were identified, and the following standards were proposed. These standards should be included in future practice in order to assist sports communities and service providers in making all processes more effective:

- it is important that first introduction tennis is more towards earlier youth with the aim of evoking positive feelings and love for the sport;
- specialization (targeted and more intensive training) is recommended later on in adolescence, as early specialization includes great risk of sports injury occurrences, experiencing great psychological stress, and consequently stopping participation in sport;
- previous research also indicates that it is desirable for tennis players, in addition to training, to do other sports as well in order to develop various skills and abilities that shall provide long-term benefits in achieving greater success in tennis.

## Data Availability

The raw data supporting the conclusions of this article will be made available by the authors, without undue reservation.
